# The impact of birth weight, birth order, birth asphyxia, and colostrum intake per se on growth and immunity of the suckling piglets

**DOI:** 10.1038/s41598-023-35277-3

**Published:** 2023-05-17

**Authors:** D. Vodolazska, T. Feyera, C. Lauridsen

**Affiliations:** Department of Animal and Veterinary Sciences, Aarhus University, Blichers Allé 20, 8830 Tjele, Denmark

**Keywords:** Immunology, Physiology

## Abstract

Colostrum is the only source of passive immunity and the major source of nutrients and is crucial for thermoregulation of newborn piglets in their early life. However, the amount of colostrum obtained by each piglet [colostrum intake (CI)] differs considerably in large litters as born by contemporary hyperprolific sow lines. This experiment aimed to investigate the impact of the following individual characteristics of piglets; birth weight, birth order and neonatal asphyxia at birth on CI, and further to determine the relationship between the CI and the passive immunity transfer, and the growth performance of piglets prior to weaning. Twenty-four Danbred sows of the second-parity and their progeny (n = 460) were used. As main inputs in the prediction model to assess individual piglet CI were piglet birth weight, their weight gain, and the duration of colostrum suckling of the piglets. The asphyxia (state of oxygen deprivation) was assessed by measuring blood lactate concentration immediately after birth, and piglets sampled at d 3 of age for determination of blood plasma concentrations of immunoglobulins (Ig) G, A, and M. Piglets’ CI was negatively associated with asphyxia (*P* = 0.003), birth order (*P* = 0.005) and low birth weight have compromised the individual CI (*P* < 0.001). Average daily gain during the suckling period was greater among piglets with high CI (*P* = 0.001) and birth weight (*P* < 0.001). Body weight at weaning (d 24 of age) was positively associated with CI (*P* = 0.0004) and birth weight (*P* < 0.001). The probability of weaning was positively associated with CI and birth weight (*P* < 0.001) of the piglets. Concentrations of IgG (*P* = 0.02), IgA (*P* = 0.0007), and IgM (*P* = 0.04) in piglets’ plasma at d 3 of age were positively associated with CI, and were negatively associated with birth order (*P* < 0.001). The present study demonstrated that piglets’ individual characteristics at birth (birth weight, birth order, state of oxygen deprivation) have considerable effects on their CI. The knowledge gained from the results of this study gives a scientific base for development and implementation of more effective techniques in practice aimed to improve the piglets’ robustness during the suckling period.

## Introduction

Colostrum intake (CI) plays a crucial role for piglets’ postnatal survival and growth providing nutrients and passive immunity^1^. Newborn piglets have limited capacity for thermogenesis as they are born with limited energy reserves^2^ and deprived of heat-producing brown adipose tissue^3^. Therefore, newborn piglets rely on the amount of energy provided through ingested colostrum. Furthermore, the colostral immunoglobulin G (IgG) is the major source of passive humoral immune protection for the piglets during the first three weeks of life^4,5^, as the epitheliochorial structure of the porcine placenta prevents the passage of maternal immunoglobulins to the foetus during the gestation period^6^. Therefore, the ingestion of an adequate amount of colostrum is crucial to ensure immune protection of the piglets. Moreover, the timing of gut closure (24 h after birth) constrains further absorption of macromolecules, as IgG from the gut lumen, compromising the transfer of maternal immunity to the piglets^7^. Thus, the early CI of newborn piglets (during the first 24 h) is necessary to ensure the absorption of adequate amounts of immunoglobulins from the colostrum.

The CI of the newborn piglets may vary considerably within and between litters due to several sow (large litter size, yield and composition of colostrum) and piglet (birth weight, birth order, birth asphyxia, viability, and onset of sucking) related factors^8^. To date, many studies have investigated piglet related factors as birth weight^9–11^ and CI^12–14^ in relation to piglet survival and growth performance. For instance, the birth asphyxia (the state of oxygen deprivation at birth) of newborn piglets and its impact on piglets’ survival and performance during nursing period has been reported elsewhere^15,16^; while the impact of asphyxiation on sufficient CI has been an object of recent research^17,18^, and relatively few studies have looked at the impact of birth order on this parameter^19,20^. In addition, the following blood parameters as albumin, glucose, lactate, pH, CO_2_, and O_2_ were reported in newborn piglets immediately after birth^21,22^. However, less attention has been paid to the individual within-litter variation of newborn piglets and the impact of such heterogeneity on CI, passive immunity transfer and subsequent growth of piglets before weaning.

Therefore, this experiment was designed to investigate the impact of the individual characteristics of piglets (in terms of birth weight, birth order, neonatal asphyxia at birth) on CI, to determine the relationship between CI and passive immunity transfer, and to explore the impact of CI on growth performance of piglets prior to weaning. This study aimed to provide the scientific basis for future management strategies in pig production, focused on improvement of robustness of piglets at weaning, and by that contribute to solving a major challenge of modern pig production i.e., the reduction of antimicrobial usage.

## Materials and methods

The experiment was performed with permission from The Danish Animal Experimentation Inspectorate, and the animal experiment was conducted according to a license obtained by the Danish Animal Experiments Inspectorate, Ministry of Food, Agriculture and Fisheries, Danish Veterinary and Food Administration. The study was carried out in accordance with the ARRIVE guidelines (Animal Research: Reporting of In Vivo Experiments).The animals used in the experiment were followed by proper veterinary surveillance throughout the experiment. Housing and rearing of the animals were in compliance with Danish laws and regulations for humane care and use of animals in research. The health of the animals was monitored daily, and illness was treated by trained personnel.

### Animals and facilities

The experiment involved 24 sows (Landrace × Yorkshire) with a congruent parity number 2 and their progeny n = 460 piglets [(Landrace × Yorkshire) × Duroc]. Piglets were born at the experimental facility at Aarhus University, AU Viborg, Research Centre Foulum. This experiment was a part of large study investigating the impact of feeding interventions of gestating sows^23^ as well as piglets performance^24^ and behaviour^25^ during suckling and early post-weaning periods. Animals were kept under controlled environmental conditions (temperature and humidity, depending on age). In the farrowing unit, sows and piglets were housed individually in pens (2.2 m × 2.4 m) with a partly slatted floor. Each farrowing crate had a creep area equipped with infrared heating lamp and floor heating to keep the temperature of the microclimate at 35 °C around farrowing. Both sows and piglets had ad libitum access to water. The ambient temperature in the farrowing room was kept at 21 °C. Sows were provided with straw as a nest building material, whereas sawdust was provided as bedding material for the piglets. All farrowings were supervised, and farrowing assistance was provided by skilled personnel if necessary.

### Experimental procedures and registrations

Immediately after birth, each newborn piglet was ear-tagged; live-born piglets were dried by paper towel; the umbilical cord was closed with a plastic strip to prevent bleeding, shortened to 10 to 15 cm, and disinfected with water-based iodine solution (10%). Birth weight, birth time, and birth order were recorded for individual piglets in the litter. Moreover, live weight of liveborn piglets was recorded again at 24 h after the onset of farrowing to determine weight gain of the piglet, which in turn was used to estimate CI of the individual piglet according to Theil et al.^26^. Litters were standardized to 15 piglets after the colostrum period, in order to meet the study design criteria of behavioural study running in parallel with the present study, which was investigating udder competition behaviour of piglets born in large litters. A live weight of 800 g was considered as a threshold value for litter standardization; thus, piglets below 800 g were euthanized. Once litters have been standardized, no piglets were moved between the litters afterwards until weaning at d 24 of age. The main criterion during the litter standardisation procedure was the following: the piglets should be kept with their own dams.

The onset of farrowing was recorded, and the average birth interval was calculated for all litters. A stillborn piglet was defined as a fully developed piglet without any signs of decay. Litter size was defined as the number of total born full-developed piglets.

### Blood and colostrum sampling, and analyses

The state of oxygen deprivation was assessed by measuring blood lactate concentration in individual piglets collected immediately after birth. During farrowing, the blood samples were collected from all newborn piglets (n = 176) in the litter with initial body weight above 800 g, however only half of all litters were sampled (n = 12) in order to minimise the number of animals exposed to painful and stressful procedures and due to practical set up in the barn. Thus, a single individual blood sample was collected within 2 min after birth from the jugular vein into a 4-mL heparinized vacutainer tube using a G 22 × 1″ 0.7 × 25 mm needle. Afterwards, the sample was drawn from the vacutainer tube into a heparinized 1-mL blood gas syringe for immediate analysis of blood lactate concentration, using RapidPoint 500 System Gas Analyzers (Siemens Healthcare Diagnostics Ltd., UK).

On d 3 *postpartum* 1 × 4-mL individual blood sample was collected from all piglets in the litter (n = 319) into heparinised vacutainers (Vacuette, Greiner Bio-One GmbH, Kremsmünster, Austria). Blood samples were centrifuged at 2000×*g* for 10 min at + 4 °C to harvest plasma samples and stored at − 20 °C until analysed for concentrations of immunoglobulins (IgG, IgM, and IgA). Concentrations of IgA, IgG, and IgM, as a representative of immune status, were analysed using a commercial kit (pig ELISA quantitation kit; Bethyl Laboratories, Montgomery TX).

Colostrum (at 0, 12, and 24 h after the onset of farrowing) and milk (on d 24 of lactation) samples were collected from the sows by hand milking. Except at 0 h colostrum sampling, an intramuscular injection of 2 mL oxytocin (10 IU/mL; Boxmeer, Holland) was used to facilitate the let-down of colostrum and milk. Approximately 50 mL of colostrum and milk was collected at each sampling time, filtered through gauze, and kept at − 20 °C until analysis. Concentrations of fat, protein, lactose, and DM in colostrum and milk were analysed in duplicate by infrared spectroscopy (Milkoscan 4000, FOSS, Hillerød, Denmark) and results are presented in Table 1. Colostrum samples collected at three sampling points were pooled together and analysed for the concentration of IgG, using a commercial kit (pig ELISA quantitation kit; Bethyl Laboratories, Montgomery TX).

**Table 1:** Analysed colostrum and milk composition.

Item	Colostrum^1^	Milk
Fat, %	6.21	6.53
Lactose, %	3.78	5.37
Protein, %	10.7	4.98
DM, %	22.6	17.5
IgG^2^, mg/mL	40.7	na^3^

^1^Colostrum samples analysed at 0 h, 12 h, 24 h, the reported values are average values of fat, lactose, protein and dry matter; milk samples analysed at d 24 *post partum*.

^2^Average IgG concentration analysed in pooled colostrum samples collected at 0 h, 12 h, 24 h.

^3^*na* not analysed.

### Statistical analyses

Statistical analyses were performed using the software R v. 4.1.2 (R Core Team, 2021) at a significance level of *P* < 0.05 and with 0.05 < *P* ≤ 0.10 declared as ‘trends’. To model possible associations between the following parameters as colostrum intake, birth weight, birth order, blood lactate concentration at birth, plasma IgG concentration at d 3 of age and growth performance data, the linear mixed models were fitted using the *nlme* package v. 3.1-153. The assumptions of normality and variance homogeneity were checked by residual plots, assisted by Shapiro Wilk’s test and by testing model reductions from models allowing for heterogeneity. A sow/litter was included as a random effect and piglets’ gender was included as fixed effect to a model. Probability of weaning was analysed by a mixed effects logistic regression using the *glmmTMB* package v. 1.1.2.3 assuming a binomial distribution where two outcomes, weaned/not weaned, at d 24 of age were defined. The visualization of adjusted predictions was performed using the *ggeffects* package v. 1.1.4. The following package computed marginal effects and adjusted predictions (or estimated marginal means) at the mean or at representative values of predictors from a defined statistical model. The result returns as data frame with consistent structure, and it was further used for creation of the plots using *ggplot2* package v. 3.4.0.

### Ethical approval

Housing and care of experimental animals complied with Danish laws and regulations for the humane care and use of animals in research [The Danish Ministry of Justice, Animal Testing Act (Consolidation Act number 726 of September 9, 1993, as amended by Act number 1081 on December 20, 1995)]. The Danish Animal Experimentation Inspectorate approved the study protocol and supervised the experiment.

## Results

### Descriptive results

The main characteristics of litters (n = 24) and total born piglets (n = 510) were recorded (Table 2). Colostrum intake per piglet averaged 356 ± 110 g (n = 460). Litter size averaged 21.3 ± 2.94 total born piglets with stillbirth occurrence of 6.57%. Birth weight of piglets averaged 1.28 ± 0.28 kg, and ranged from 476 to 2073 g and within litter birth weight variations ranged from 155 to 361 g. The birth interval averaged 17 ± 26 min. The number of male live-born piglets 248 (48.8%).

**Table 2:** Litter characteristics, reported values are averages ± SD for 24 litters used in the experiment.

Item	Value
Number of piglets in colostrum study (n)	460
Colostrum intake (g)	356 ± 110
Birth weight (kg)	1.28 ± 0.28
Total litter size at birth^1^ (n)	21.3 ± 2.94
Stillbirth (%)	6.57
Birth interval (min)	17 ± 26
Gender (%)	
Male	48.8
Female	51.2

^1^Total litter size at birth is the sum of liveborn and stillborn piglets.

### Piglets’ individual parameters at birth

**Figure 1:**
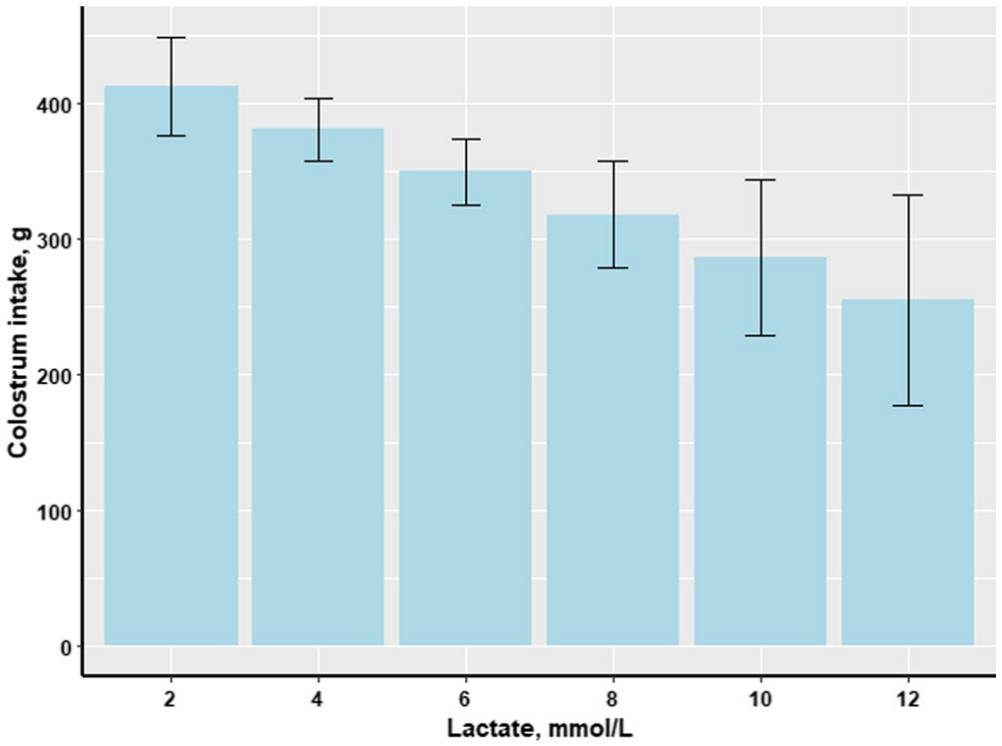
Effect of piglets’ blood lactate concentration (mmol/L) at birth on colostrum intake (g) of piglets (n = 176).

**Figure 2:**
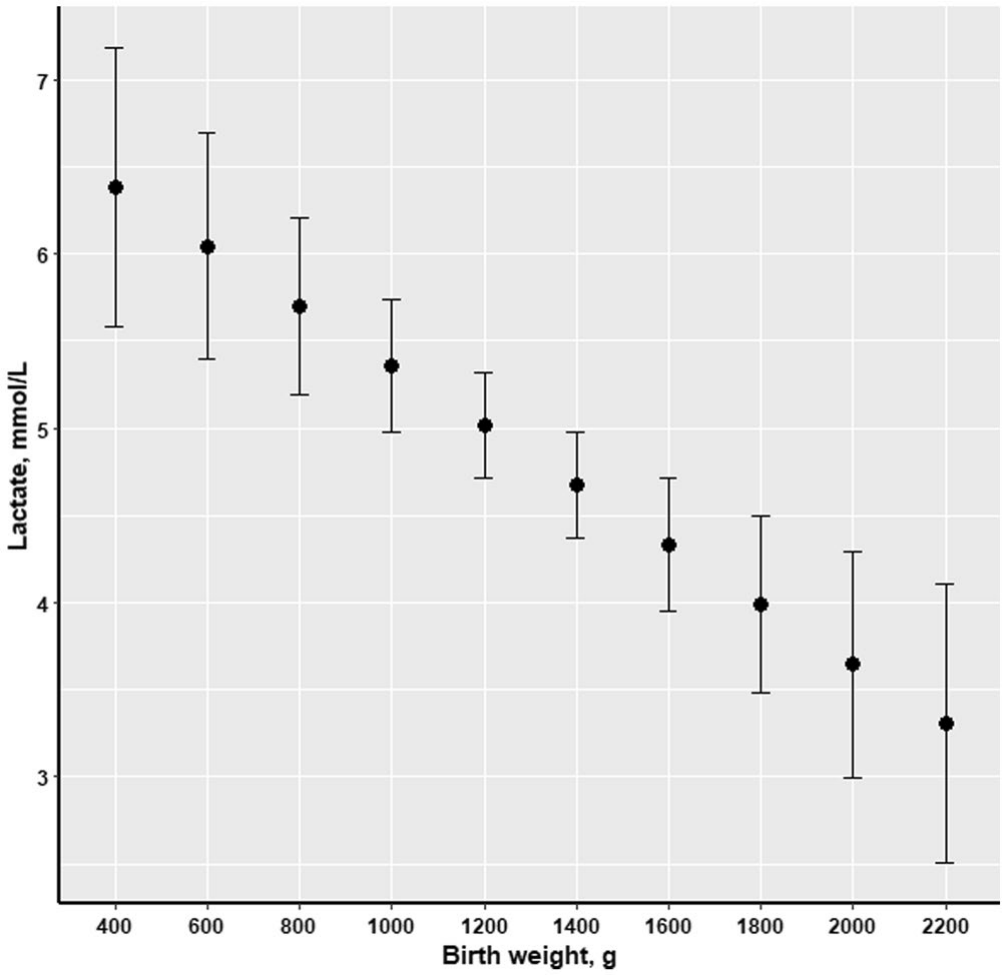
Effect of piglets’ birth weight on blood lactate concentration (mmol/L) of the piglets at birth (n = 176).

**Figure 3:**
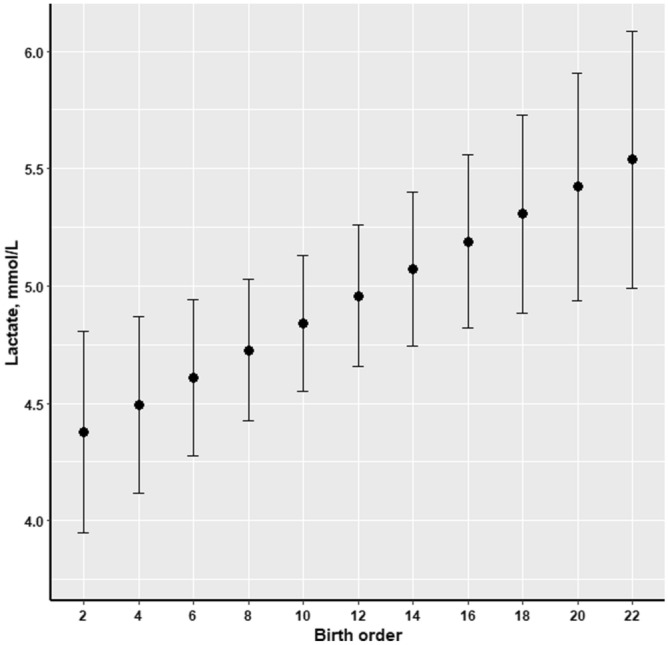
Effect of birth order on piglets’ blood lactate concentration (mmol/L) at birth (n = 176).

Piglets’ CI was negatively associated with blood lactate at birth (*P* = 0.003; Fig. 1), thus, newborn piglets with high levels of blood lactate had a low CI and the same association was observed in relation to piglets’ birth weight (*P* = 0.004; Fig. 2). The opposite pattern was observed with regard to lactate concentration and birth order, as blood lactate increased with increasing birth order of the piglets (*P* = 0.005; Fig. 3).

**Figure 4:**
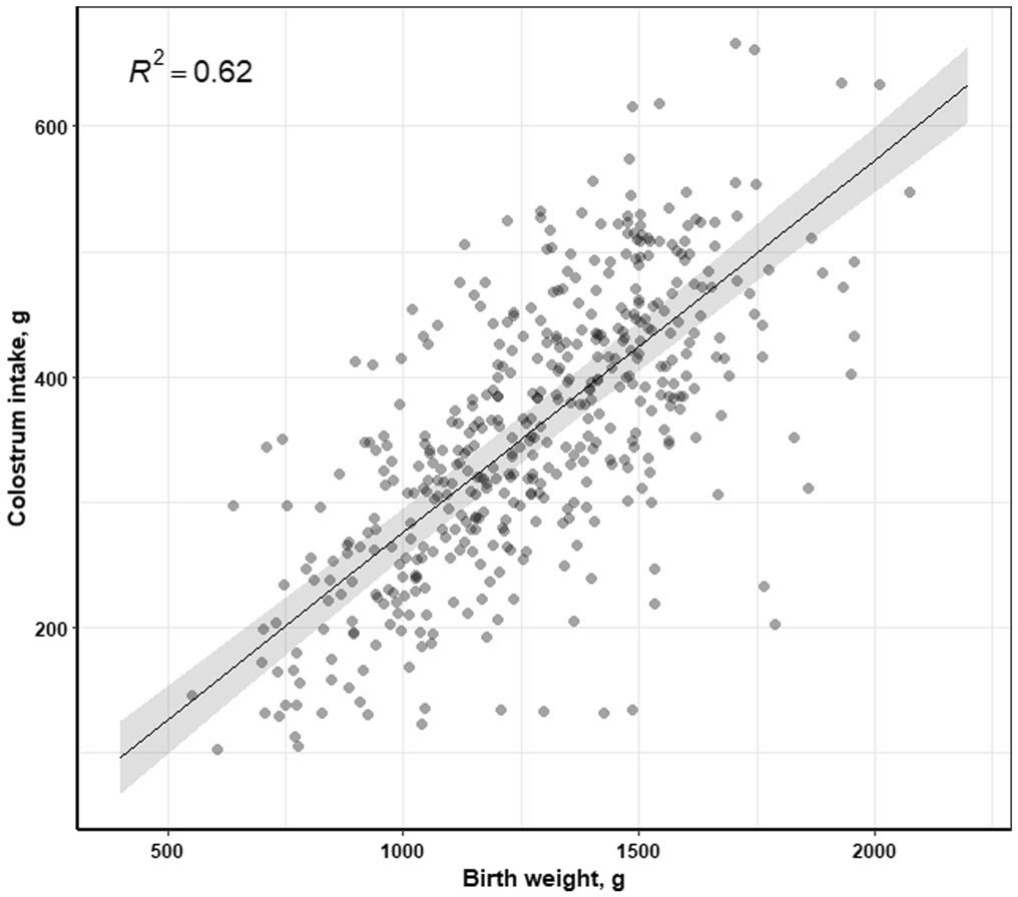
Effect of piglets’ birth weight (g) on colostrum intake (g) of the piglets (n = 460).

CI was positively correlated with piglets’ birth weight (*P* < 0.001, R^2^ = 0.62, Fig. 4), i.e., piglets with greater birth weight ingested considerably more colostrum compared to their littermates with lower birth weight. In addition, CI was negatively correlated with birth order (*P* = 0.0001, data not shown), as amount of ingested colostrum decreased with increased birth order.

### Piglets’ growth performance

**Figure 5:**
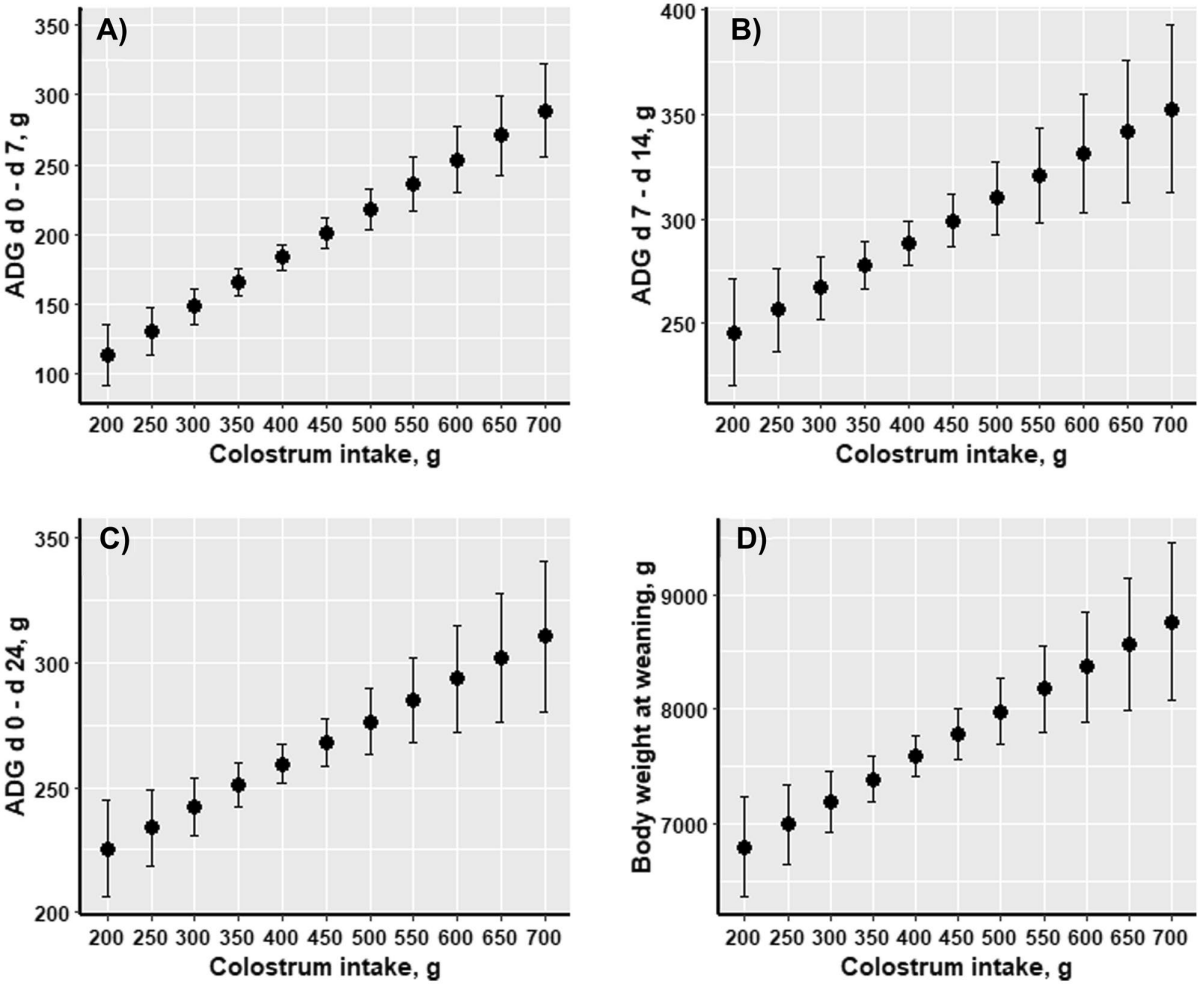
Effect of colostrum intake on average daily gain and on weaning weight of the piglets. Graph (**A**–**C**) represent the impact of individual colostrum intake (g) on average daily gain (ADG, g) during the suckling period; graph (**D**) represents the impact of individual colostrum intake on weaning weight of the piglets (n = 360).

Average daily gain during the suckling period was significantly influenced by CI and birth weight (Fig. 5). Thus, piglets with greater CI (*P* = 0.001) and greater birth weight (*P* < 0.001) gained more weight in average on a weekly basis when compare to their littermates with low CI and low birth weight. Body weight at weaning was positively associated with CI (*P* = 0.0004) and birth weight (*P* < 0.001).

**Figure 6:**
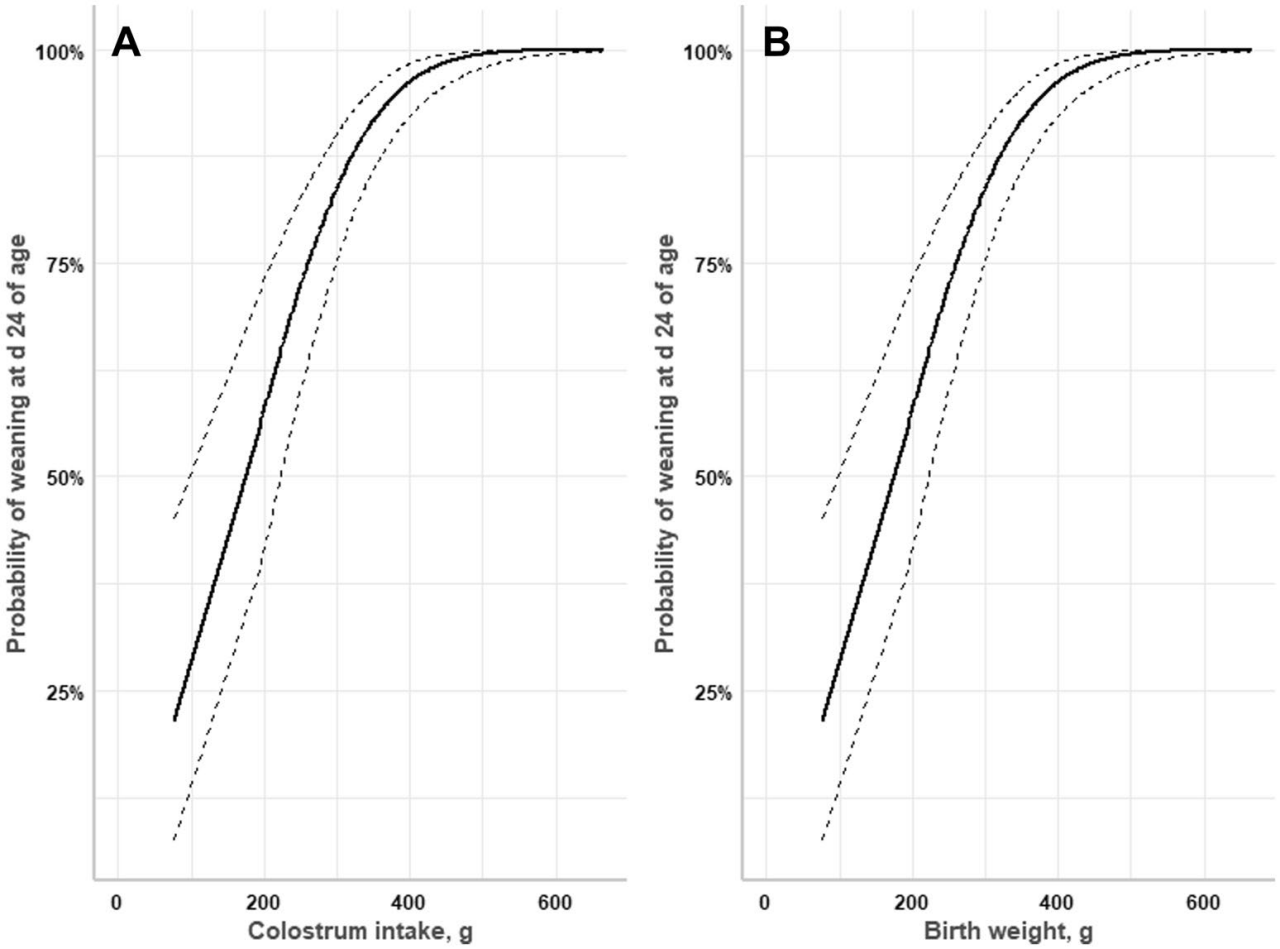
Effect of individual colostrum intake (g) and birth weight (g) on the probability of weaning**.** Graph (**A**) represents the effect of individual colostrum intake (g) of piglets and graph (**B**) represents the effect birth weight on the probability of weaning at d 24 of age (n = 360).

In addition, CI and birth weight were positively associated with the probability of weaning (*P* < 0.001, Fig. 6). Thus, piglets with CI over 400 g had 100% probability to be weaned.

### Assessment of passive immunity transfer

**Figure 7:**
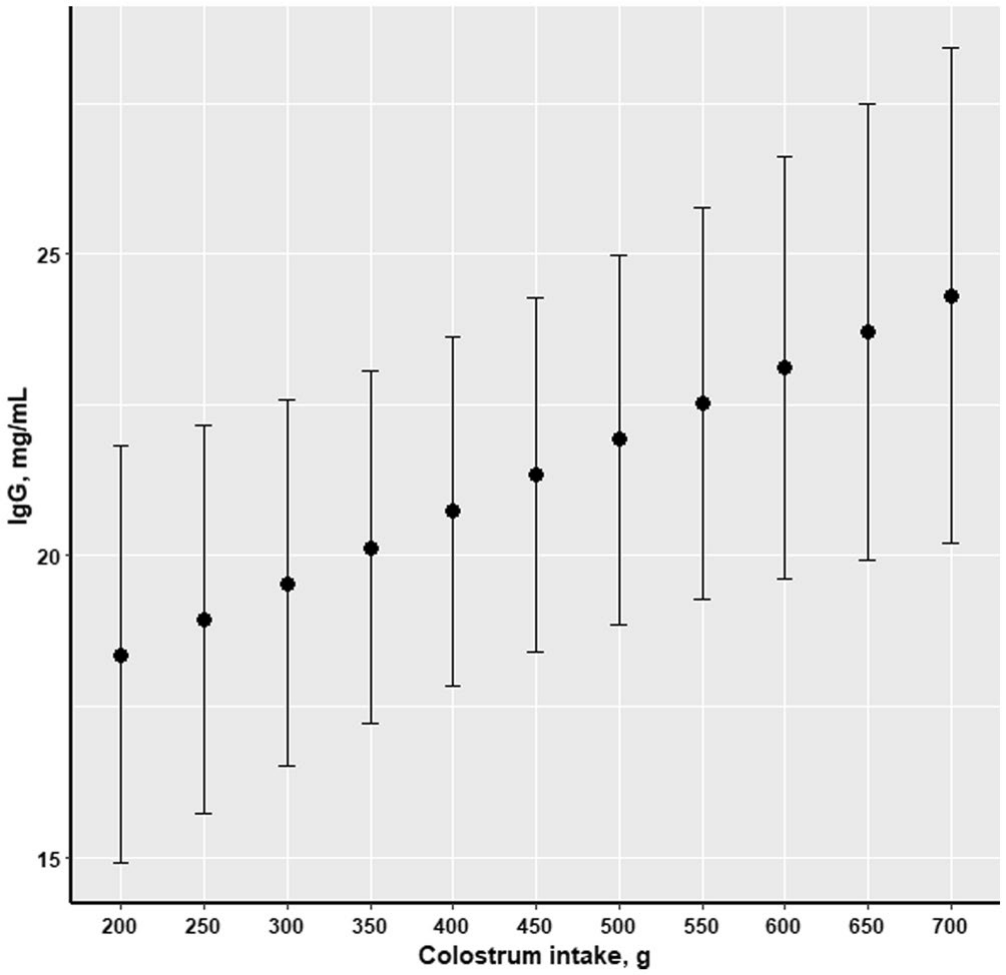
Effect of piglets’ colostrum intake on plasma IgG concentration (mg/mL) at d 3 of age (n = 319).

**Figure 8:**
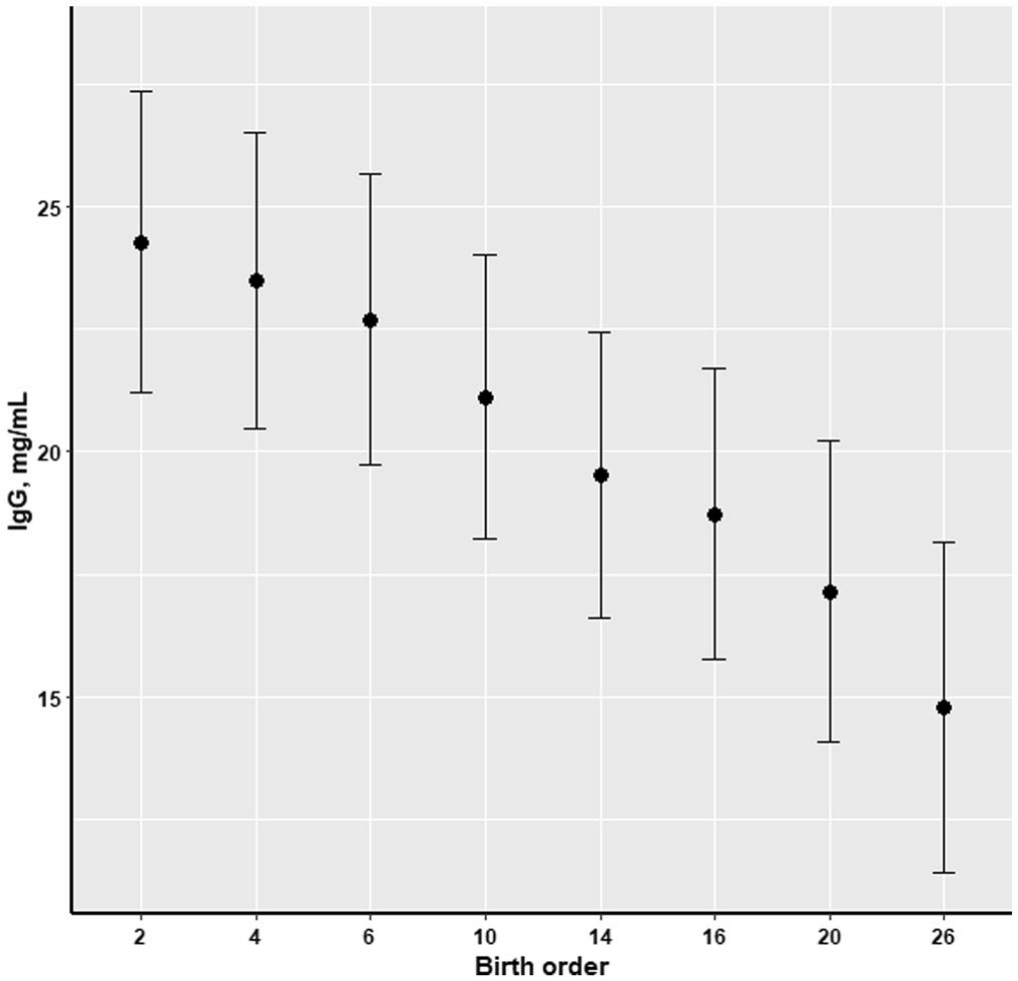
Effect of birth order on piglets’ plasma IgG concentration (mg/mL) at d 3 of age (n = 319).

The amount of ingested colostrum was reflected in the plasma IgG concentration measured at d 3 of age (Fig. 7). Thus, piglets characterised by having high CI had higher concentration of plasma IgG at d 3 of age, compared to those with low CI (*P* = 0.02); while birth weight had no influence (*P* = 0.33) on plasma IgG at d 3 of age. Furthermore, concentration of IgG at d 3 of age was negatively associated with birth order (*P* < 0.001), as this parameter decreased when birth order increased (Fig. 8), and such relationship to birth order was also observed for plasma concentrations of IgA (*P* = 0.0007) and IgM (*P* = 0.04) in piglets of 3 days of age (data not shown).

## Discussion

Individual colostrum intake of newborn piglets is a major constraining factor of postnatal survival of piglets in modern pig production. Adequate colostrum intake is a challenge due to large variation of the colostrum production between sows, and the intake per se varies greatly between littermates. The practice of breeding towards large litter size has resulted in an increased number of low birth weight piglets with less viability in the litter, meanwhile colostrum intake per individual piglets decreased substantially over the past decades^27^. Thus, high within-litter heterogeneity induces the direct competition for udder among littermates during the first 24 h *postpartum* resulting in exclusion of lightweight littermates from functional and productive teats by their heavyweight littermates. In addition, the larger littermates stimulate and drain the best productive teats more effectively, consequently ingesting more nutrients and other essential compounds from the colostrum and milk^28,29^. Consequently, the low body weight at birth has negative impact on piglets’ survival and growth throughout lactation^28^. Moreover, low birth weight piglets are physiologically compromised in terms of energy reserves at birth^8^ and more susceptible to asphyxia during farrowing due to lower oxygen pool in their body^30^. In agreement with the latter findings^18,31^, the present results demonstrated a strong negative correlation between piglet birth weight and blood lactate concentration at birth, documenting that low birth weight piglets are at high risk of birth asphyxia as compared with their heavier littermates. Larger litter size at birth resulted in prolonged farrowing duration and lower viability of the liveborn piglets because of state of oxygen deprivation and delayed first suckling^22,32^. In line with this, our results clearly revealed that piglets with higher concentration of blood lactate at birth had lower colostrum intake, as increased levels of blood lactate indicates a state of oxygen deprivation (asphyxia) of newborns^33^. It indicates that the asphyxia state depressed viability and suckling activity of the piglets, which is in agreement with previously reported^15^. Additionally, in agreement with prior findings^31,32^ the results of the present experiment have demonstrated that last-born piglets (born in the later phase of parturition) have suffered more from asphyxia compared to first-born piglets (born at the early phase of parturition). This result demonstrates a major challenge of modern pig production in terms of hyperprolific sows as litter size has increased at the expense of the number of physiologically less mature and low weight piglets, and in parallel an increase of the length of parturition. Thus, the later-born piglets may suffer more from asphyxiation, as they experience the cumulative effects of successive contractions with consequent reducing the level of oxygenation of the piglets^34^. This can potentially lead to severe hypoxic ischaemic organ damage in newborns, followed by a fatal outcome or severe life-long pathologies^35^. Thus, prolonged moderate in-utero asphyxiation weakens piglets and renders them less capable of adaptation to *extra-uterine* life^15^. The findings of this experiment complement those of earlier studies and highlight the necessity of the development of novel strategies improving the survival and growth of asphyxiated piglets. In this context, the oxygen supply of neonatal piglets is one of the promising strategies reported in the literature; e.g., the recent study of Soraci et al.^36^ have demonstrated that oxygen supply improved physiological and productive parameters of piglets born with signs of asphyxia or very low birth weight. However the study of Vande Pol et al.^37^ have revealed that drying of piglets and oxygen supply provided no additional benefit over drying alone, which is contradicting to what reported of Soraci et al.^36^ and calling for more research.

The present study has furthermore demonstrated the strong relationship between colostrum intake and passive immunity transfer from sows to offspring. As it was previously emphasized in the literature, the major part of the total immunoglobulins present in sows colostrum is IgG^38^, thus plasma IgG concentration of the piglets on d 3 of age was used in this study as a biomarker for evaluation of the transfer of passive immunity from sows to piglets. Consequently, the present results indicated a strong positive association between CI and plasma IgG concentration of the piglets, which is consistent with earlier reports^2,20^. Furthermore, this result suggests that plasma IgG concentration obtained in piglets 3 days after birth provides valuable data for quantification of the IgG uptake into circulation of the piglet before intestinal closure^39^. As it was previously emphasized in the literature, the intact protein molecules as IgG cannot be absorbed after intestinal closure, 24–36 h after birth^4^. Thus, the IgG measured in the plasma of piglets 3 days after birth originated from colostrum. Therefore, plasma IgG concentration measured in early life could be applied as a direct measure of efficiency of passive immunity transfer from the dam to offspring, and it could potentially have a great practical application. However, birth order was negatively associated with plasma IgG concentration of the piglet on d 3 of lactation. This result indicates that the last-born piglets had ingested less and lower quality colostrum (in terms of passive immunization) when compared to their littermates born within the early phase of parturition. Moreover, farrowing duration increase along with litter size, and this could have a confound effect on the impact of birth order on IgG concentration of the piglets. This is due to the fact that two-thirds of the total volume of colostrum is secreted by the udder within 12 h after birth of the first piglet^40^, while colostral IgG declines abruptly within the first 6 h after the onset of parturition^41^. Considering the importance of this result for the practice, it indicates that piglets born late in the birth order experienced a lower rate of passive immunity transfer.

This study was restricted to the Danbred genetic line, which is the most commonly used genetic line in the Danish pig industry. However, other studies, such as the one conducted by Declerck et al.^42^ have compared four commercial crossbred lines (PIC, Topigs, Hypor and Danbred). Their findings suggested that piglets’ colostrum intake was affected by breed, while colostrum yield did not differ significantly between breeds. Thus, Declerck et al.^37^ has claimed that the association between breed and colostrum intake besides litter size was affected by other breed factors, such as udder morphology or teat access. However, in the present study neither assessment of the sow-associated factors was performed nor interrelationship between sow- and/or piglet-associated factors was investigated. The results obtained in our study emphasize the significance of piglet-related factors and their connection to individual colostrum intake, and explore the impact of CI on piglets’ immunity and performance before weaning. Nevertheless, comparison between different genetic lines with respect to piglet related factors could be suggested as topic for further research; but it was not possible to accommodate more genetic lines in this study.

The present study demonstrated a strong positive association between CI and birth weight of the piglets as well as subsequent growth performance of the piglets until weaning on d 24 of age. Similar findings were reported, previously emphasizing the importance of heavy birth weight and high CI on pre-weaning growth performance of the piglets^9–11,13^. Additionally, we observed the positive impact of CI on piglets’ survival and daily gain prior to weaning, which was in agreement with previously obtained results^2,12,14^.

## Conclusion

The present study has demonstrated that piglets with low birth weight and born late in the birth order were more prone to neonatal asphyxia, which is a factor hampering CI.

The low CI and high birth order number influenced negatively passive immunity transfer from sows to piglets; while high CI and low birth order number improved the plasma immunoglobulin concentrations at d 3 after birth.

The robustness at weaning in terms of weaning weight was found to be strongly associated with CI and birth weight. Considering that modern pig production is struggling with finding solutions to reduce antibiotic usage, the present study demonstrated that the adequate CI during the first 24 h of life is crucial for proper immune transfer from sows to offspring, and by that may enhance the resilience of piglets to pathogens at weaning. Our results emphasise that future management strategies directed to the improvement of piglets’ individual characteristics at birth could be beneficial for improvement of piglets’ overall robustness prior to weaning, and this will improve piglets’ resilience to pathogens during the early post-weaning. Hence, it could reduce the usage of antimicrobials in pig production. The knowledge gained with the results of this study gives a scientific base for development and implementation of strategies in practice aimed at effective management techniques to improve piglets’ survival, performance, immunity and robustness during the suckling period.

## Data Availability

None of the data sets were deposited in an official repository. The data that support the study findings are available upon request per mail to corresponding author Darya Vodolazska dvs@anivet.au.dk.

## Abbreviations

ADG: Average daily gain
CI: Colostrum intake
ELISA: Enzyme-linked immune sorbent assay
IgA: Immunoglobulin A
IgG: Immunoglobulin G
IgM: Immunoglobulin M
